# Virtual Evolution of HVEM Segment for Checkpoint Inhibitor Discovery

**DOI:** 10.3390/ijms22126638

**Published:** 2021-06-21

**Authors:** Mingjia Yu, Huimin Zhao, Yuhui Miao, Shi-Zhong Luo, Song Xue

**Affiliations:** 1 Beijing Advanced Innovation Centre for Soft Matter Science and Engineering, Beijing University of Chemical Technology, Beijing 100029, China; 2019750009@mail.buct.edu.cn; 2 Beijing Key Laboratory of Bioprocess, College of Life Science and Technology, Beijing University of Chemical Technology, Beijing 100029, China; 18634404469@163.com (H.Z.); ukimiao@foxmail.com (Y.M.)

**Keywords:** BTLA, virtual screening, peptide inhibitor, immune checkpoint

## Abstract

Immune therapy has emerged as an effective treatment against cancers. Inspired by the PD-1/PD-L1 antibodies, which have achieved great success in clinical, other immune checkpoint proteins have drawn increasing attention in cancer research. B and T lymphocyte attenuator (BTLA) and herpes virus entry mediator (HVEM) are potential targets for drug development. The co-crystal structure of BTLA/HVEM have revealed that HVEM (26–38) fragment is the core sequence which directly involved on the interface. Herein, we conducted virtual evolution with this sequence by using saturation mutagenesis in silico and mutants with lower binding energy were selected. Wet-lab experiments confirmed that several of them possessed higher affinity with BTLA. Based on the best mutant of the core sequence, extended peptides with better efficacy were obtained. Furthermore, the mechanism of the effects of mutations was revealed by computational analysis. The mutated peptide discovered here can be a potent inhibitor to block BTLA/HVEM interaction and its mechanism may extend people’s view on inhibitor discovery for the checkpoint pair.

## 1. Introduction

Immunotherapy has drawn increasing attention as novel cancer therapy which shows great clinical significance in recent years [1,2]. Immune-checkpoint inhibitors (ICI) have made great progress as a crucial component of immunotherapy [3]. Antibodies targeting cytotoxic T-lymphocyte antigen-4 (CTLA-4) and programmed death-1/ligand (PD-1/PD-L1) have increasingly become new standards of care in several types of cancers [3,4,5]. ICI is a set of molecules that regulate the immune system and can stimulate or suppress the immune response [6,7,8]. To maintain proper immune balance, the body activates the immune system to fight foreign bodies through a series of stimulating pathways, and avoids the damage of excessive activation to normal tissues through inhibitory pathways, such as CTLA-4 pathway [9,10]. However, cancer cells can suppress the immune response by abnormally expressing inhibitory checkpoint receptors to escape the killing effect dominated by the T cell response, such as PD-1 [11,12,13]. Thus, the immune system can be re-activated to kill cancer cells by blocking these immunosuppressive pathways [7,14]. In addition to the above pathways, other immune-checkpoints and their ligands could also serve as targeted [4]. One of these is the B- and T-lymphocyte attenuator (BTLA) [15,16,17], which is abundantly expressed on naive human CD8 + T cells but downregulated upon CD8 differentiation [18,19]. However, BTLA remains in high level in some tumor specific CD8 + T cells like melanoma and continue acting as negative immune regulator upon the herpes virus entry mediator (HVEM) binding [16,20,21]. Hepatitis B infection can also result in high expression of BTLA in virus-specific T-cells, attenuating immune response to the virus [19,22].

Inspired by the success of blocking PD-1/PD-L1 interaction for immunotherapy [14,23,24,25,26,27], inhibition of BTLA/HVEM binding showed potential for the treatments of infectious diseases and cancers. Antibodies against BTLA has been shown to reverse the inhibition of T cells [16].

In recent years, peptides have displayed excellent potential as inhibitors targeting protein–protein interactions (PPIs) [25,28]. Compared with small molecules [29], peptide drugs can form a larger binding interface with target proteins, so they have better specificity and binding ability, and are more suitable for PPIs [30]. Therefore, it is of great significance to study peptide inhibitors for the next generation of immune checkpoint based anticancer drugs. Especially for the BTLA/HVEM system with more complex interaction [2,31,32,33], peptide inhibitors have better regulation in the selection of specific interfaces. Fortunately, the crystal structure of BTLA/HVEM complex has been reported (PDB: 2AW2), providing a structural basis for the development of inhibitors [34]. The HVEM (26–38) peptide was shown to be directly involved in the binding of BTLA [35]. Peptides containing this core sequence had been confirmed to block the ligation between BTLA and HVEM [35,36].

Herein, we would like to explore if mutations in the core sequence (26–38) could lead to better affinity. Single-point saturation mutagenesis of the 13 amino acids were simulated using DISCOVERY STUDIO v4.5 computer program to improve affinity, with each amino acid mutated into other 19 different amino acids. Those mutants can give us structure–activity relationship information on the interface and help to design inhibitors, which possess lower binding energy. According to the results of computational simulation, single and double mutants of the core sequence with the lowest binding energy were synthesized (Table 1) and their relative affinity with BTLA was analyzed by competitive ELISA. A double mutant was picked, showing the highest affinity with BTLA. Though the core sequence (26–38) was shown to directly involved in the interface, longer peptides were usually used for inhibitor design [36]. Thus, extended peptides (17 and 26 aa) based on the core sequence mutant were synthesized for the evaluation as inhibitors. As expected, both peptides displayed better affinity with BTLA. The binding mechanism between the peptide and BTLA was revealed by simulation by ZDOCK protocol with DISCOVERY STUDIO [37].

## 2. Results and Discussion

### 2.1. Saturation Mutagenesis of HVEM(26–38) In Silico

**Figure 1:**
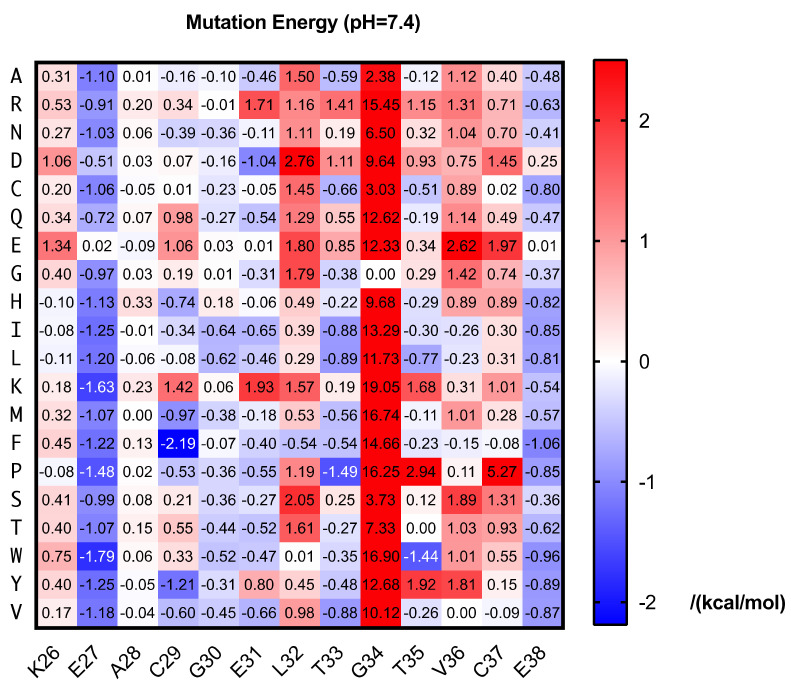
Predicted mutation energy of BTLA-HVEM (26–38) binding at pH 7.4.

Mutations are the raw materials of evolution. We aim to obtain peptide inhibitors evolved from HVEM segment, which possesses a higher affinity than the natural protein. Since the HVEM (26–38) has been reported to directly interact with BTLA, mutagenesis on this sequence is likely to result in peptides as competitive inhibitors of BTLA/HVEM interaction. Though a short sequence it is, screen every possible mutation is a great challenge for experiments. Thanks to the rapid advances of computational chemistry, we are allowed to do saturation mutagenesis by simulation. Each amino acid in the HVEM (26–38) was mutated to other 19 amino acids and the binding energy was calculated for each peptide-BTLA complex by DISCOVERY STUDIO. The difference from the native one is shown as mutation energy here (Figure 1).

The results showed abundant mutations resulted in lower mutation energy, indicating potential better binding affinity. E27 and E38 seem to be disfavored for the binding and most mutations on these two sites gave lower energy. We chose the mutants with lowest mutation energy for each amino acid and set a threshold of −1 kcal/mol for our wet-lab screening library. Additionally, we chose the mutants on E27 and E38 with the mutation energy below −1 kcal/mol and calculated the combined mutation energy (Figure S1). Three best double mutants predicted were selected. In total, 12 mutated peptides—including 9 single mutants and 3 double mutants together with the wild-type—were synthesized for competitive ELISA screening (Table 1).

### 2.2. Screening of Peptide Inhibitors

**Figure 2:**
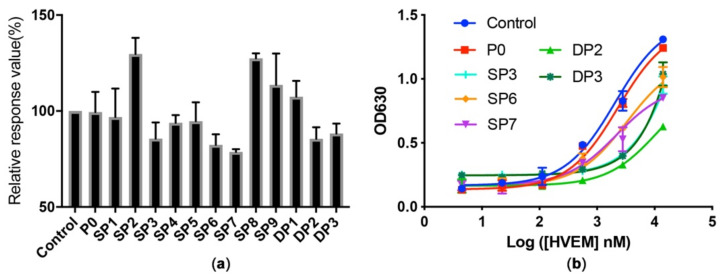
Screen and characterization of predicted peptides. (**a**) Preliminary screen with 1 mg/mL peptides by competitive ELISA. (**b**) Titration of selected peptides-BTLA binding with different concentrations of HVEM.

Though computational methods provided certain guidelines for picking the mutations, they are not 100% accurate since simplified models were used, like ignoring the possible conformation changes caused by mutations. Thus, the wet-lab examination is necessary to confirm their inhibition effects. The library containing predicted peptides was synthesized by solid phase peptide synthesis (SPPS) method with Fmoc strategy. We used competitive ELISA for the screen to ensure the positive peptides selectively binds in situ on the BTLA/HVEM interface. Biotin-HVEM was used as the signal reporter. Peptides with strong binding of BTLA will compete with Biotin-HVEM and result in lower signal. A primary screen was carried out by using 1 mg/mL of each peptide and those with response value under 85% of the control were selected for further characterization (Figure 2a).

Three single mutants (SP3, SP6, and SP7) and two double mutants (DP2 and DP3) were selected and dose dependent competition assays were conducted. Different concentrations of HVEM were used to titrate peptide-BTLA complexes (Figure 2b). Clearly, the double mutant DP2 showed the best inhibitory effects for BTLA/HVEM binding.

### 2.3. Design and Evaluation of Extended Peptides Based on DP2

**Figure 3:**
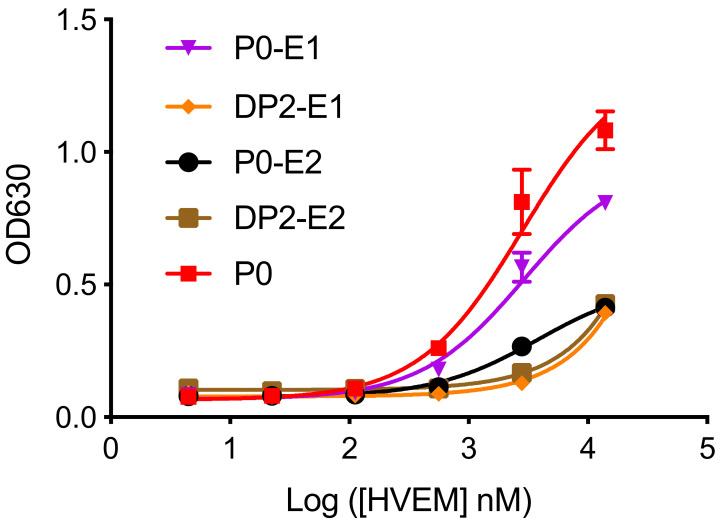
Titration curve of extended DP2 or P0 with HVEM.

DP2 showed certain effect to block BTLA/HVEM binding. However, the affinity seems to be low from the titration curve. Interestingly, the original fragment from HVEM, namely P0 here, demonstrated little inhibition. Though identified as the core sequence for binding, existing reports used extended peptides HVEM (23–39) and (14–39) for inhibition studies, which strongly suggested that terminal residuals are critical for binding. Thus, we extended DP2 to match the reported length of the natural fragments, affording DP2-E1 (Ac-YRVKPACGELTGTVCFP-NH_2_) and DP2-E2 (Ac-ESCPKCSPGYRVKPACGELTGTVCFP-NH_2_). We would like to explore if the extension gives better affinity. The two peptides with the natural version (P0-E1 and P0-E2) were synthesized for comparison (Figure 3). Not surprisingly, P0-E1 and P0-E2 both showed more potency than P0, with the longer one the better. The extended mutants, DP2-E1 and DP2-E2 demonstrated a higher affinity than the native ones. The surface plasmon resonance (SPR) was used to further examine the affinity of the peptides quantitatively (Table 2 and Figure S1). The data confirmed that P0 can barely bind to BTLA but DP2 possesses certain affinity with BTLA with *K_D_* of micromolar. The longer P0-E1 has a lower *K_D_* of 9 mM compared with P0. The mutant DP2-E1 binds slightly tighter than P0-E1, however weaker than DP2. The longest DP2-E2 showed highest affinity with a *K_D_* as low as 1.46 nM, which is much better than the original form of P0-E2.

### 2.4. Mechanism of Action of the Peptides Predicted by Docking

**Figure 4:**
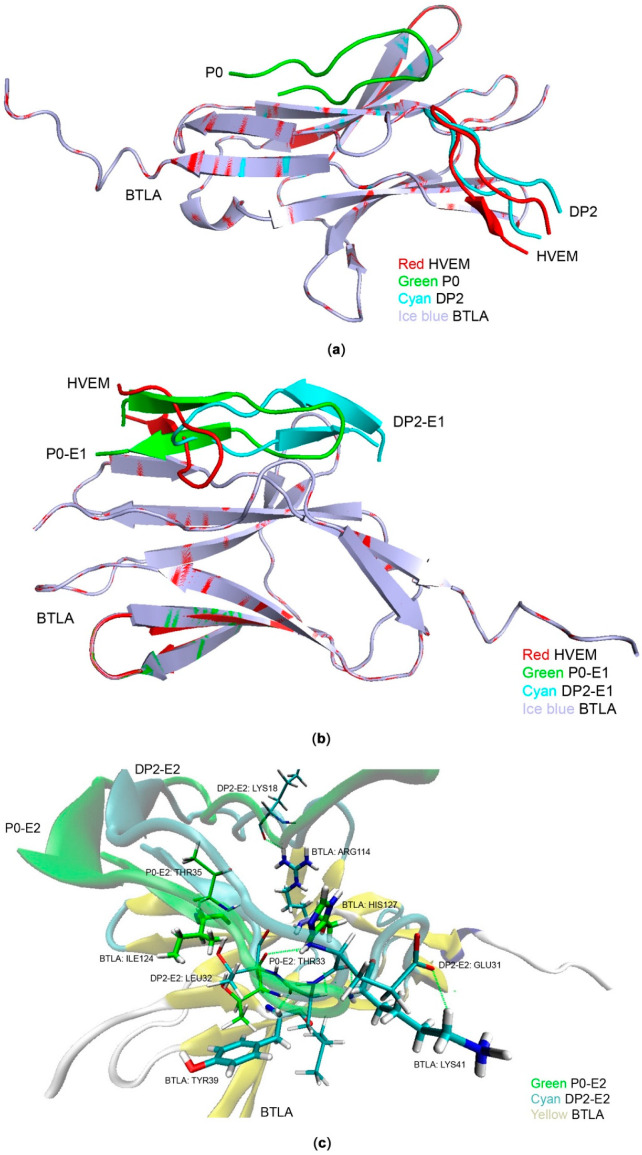
Docking results showing the predicted interactions between the native or mutated peptides and BTLA. (**a**) P0 binds to a different site on BTLA from HVEM but DP2 binds on the same site as HVEM. (**b**) P0-E1 and DP2-E1 bind on the same site as HVEM but with various orientation. (**c**) P0-E2 and DP2-E2 bind at the same site on BTLA, the latter with more hydrogen bonds formed.

To explore the mechanism for the differential affinity between the peptides and BTLA, we analyzed the interactions by protein–protein dockings using Dock Proteins (ZDOCK) protocol in DISCOVERY STUDIO [38]. Overlay of the complexes shows that the peptides of HVEM/P0/DP2 (red/green/cyan) binding to BTLA (ice blue) having huge positional difference (Figure 4a), while P0 (green) is shifted far away from the other two peptides in the BTLA molecule and DP2 (cyan) has kept in the similar site as HVEM. The other models for complexes of interest docked to BTLA (BTLA + P0-E1, BTLA + DP2-E1, BTLA + P0-E2, BTLA + DP2-E2, BTLA + SP1, BTLA + SP2, BTLA + SP3, BTLA + SP4, BTLA + SP5, BTLA + SP6, BTLA + SP7, and BTLA + DP1 mentioned in the text) were provided in Supplementary Materials (Figure S2). In some of these models, including P0, the peptides showed ‘off-site binding’, indicating weak binding affinity on the BTLA/HVEM interface, explaining their weak inhibitory effects. According to the overlay results of the complexes, it reveals that P0 lacks the strong hydrogen-bond interactions with BTLA, resulting in no valid binding sites of BTLA, while DP2 interacts well with BTLA. It is also interesting to note that after the truncations of the core sequence (26–38) from HVEM in the theoretical models, both P0 and its double mutant-DP2 have changed the secondary structure compared with HVEM segment.

Overlay of the complexes shows that the peptides of HVEM/P0-E1/DP2-E1 (red/green/cyan) binding to BTLA (ice blue) having minor positional difference (Figure 4b). Based on the position of HVEM, P0-E1 (green) and DP2-E1 (cyan) have changed angles a bit in the binding sites of BTLA molecule. The results of SPR concludes that the values of binding affinity between BTLA and P0-E1/DP2-E1 are close, which suggests that the orientation of peptides would not play a pivotal role in affecting the interaction on the interface between BTLA and its ligands; however, the huge conformational transformation would.

To further elucidate the binding modes between BTLA (yellow) and P0-E2/DP2-E2 (green/cyan) (Figure 4c), we examined the docking complexes drawn by 3D molecular structures. The terminals of the two peptides have similar conformation while a conformation change occurred for DP2-E2 in the middle part, resulting in more interactions. In detail, the 3D models show that the amino acids of Ile124, His127, Arg114, Tyr39, and Lys41 on the surface of BTLA form multiple hydrogen bonds with amino acid residues of Thr35 and Thr33 in P0-E2, while Lys18, Leu32, and Glu31 in DP2-E2. The discrepancy on the number of hydrogen bonds between BTLA and P0-E2/DP2-E2 leads to the conclusion that more hydrogen bonds correspond to a higher binding affinity. Above all, DP2-E2 is the best inhibitor interacting in the binding sites of BTLA with the most hydrogen bonds, compared to DP2 and DP2-E1 (Tables S1–S4).

The docking provided the structure basis of the binding mechanism of the native and mutated peptides. However, the method is rough in theory since the model is relatively rigid. To justify whether the models constructed from protein–protein docking are reliable, the molecular dynamics (MD) simulations were performed. Both methods gave similar complex structures (Figure S3). Quantitatively, the RMSD values of the structures from the MD and ZDOCK are small (Figure S4), which indicated that the structural variance is minor for the two methods. We believe that P0 and DP2 do have different binding sites and the more hydrogen bonds contributed to the higher affinity of DP2-E2 compared with P0-E2 due to the conformation changes.

## 3. Materials and Methods

### 3.1. Calculate Mutation Energy (Binding)

The crystal structure of BTLA/HVEM complex (A and B chains) was obtained from PDB (Protein Data Bank ID: 2AW2). The PDB files were cleaned and the heteroatoms (HETATM) of the structure were removed by CHARMM-GUI (Dr. Im’s research team, Philadelphia, PA, USA, http://www.charmm-gui.org (accessed on 23 April 2021)) [39]. The HVEM (23–39) peptide has already been confirmed that it can block BTLA/HVEM ligation. Thus, to obtain peptides (based on HVEM (23–39) peptide) which bind more strongly to BTLA, the single-point saturation mutagenesis of each 13 amino acids on HVEM (26–38) peptide were performed by Calculate Mutation Energy (Binding) protocol in DISCOVERY STUDIO v4.5 (Dassault Systemes, BIOVIA Corp., San Diego, CA, USA, (accessed on 19 May 2021)) computer program to evaluate the effect of mutations on the binding affinity of molecular partners in BTLA-HVEM complexes. The energy effect of each mutation on the binding affinity (mutation energy, ΔΔG_mut_) is calculated in CHARMm Polar H force field and pH-dependent mode. The pH value, ionic strength, solvent dielectric constant, and energy cutoff were set at 7.4, 0.1, 80, and 0.5, respectively. The mutation energy represents the stability of the BTLA-HVEM complex, in other words, the lower mutation energy indicates the more stable structure of the BTLA-HVEM complex [37].

### 3.2. Peptide Synthesis

Peptides were synthesized by Jiangsu Shenlang Biotech Co., Ltd. using solid phase peptide synthesis (SPPS) method using Fmoc strategy.

### 3.3. Competitive ELISA

96-well EIA/RIA plates (High Binding, Corning Incorporated) were coated with 350 ng/well of recombinant BTLA-Fc protein in PBS (50 µL/well), incubating at 37 °C overnight until the solutions evaporate completely. Methanol was added for protein fixation and the wells were blocked with 5% BSA in PBS (200 µL/well). The peptides in DMSO were diluted into 1 mg/mL in PBS (50 µL/well) and incubated 12 h at room temperature. Wells were washed six times with PBST (phosphate buffer saline with 0.1% Tween-20, pH 7.4). Different concentrations of recombinant HVEM-biotin protein (50 µL/well) was added and incubated for 2 h at room temperature. Wells were washed six times with PBS. Horseradish peroxidase (HRP) labeled Streptavidin was finally added at a 1/5000 dilution in PBS and incubated for 2 h at room temperature. 3, 3′, 5, 5′,-tetramethylbenzidine (TMB, Beyotime) was used for color development. The absorbance was read at 630 nm using Microplate Reader. Statistical analysis of the results was performed using GraphPad Prism 7 software.

### 3.4. Surface Plasmon Resonance

The SPR assay was conducted using Biacore T100 system with Sensor Chip CM5 (Cytiva, Marlborough, MA, USA). BTLA-Fc was conjugated to the chips with EDC/NHS coupling in NaAc buffer. The assay was performed in PBS buffer with 2% DMSO. The buffer calibration was conducted by mixing different volume of buffers with 1.5% or 2.8% DMSO. Various concentrations of the peptides were injected and flow through the chip at 30 µL/min. The signals were recorded and analyzed with either static (for fast dissociation) or kinetic (for slow dissociation) model.

### 3.5. Dock Proteins (ZDOCK)

The protein–protein dockings were conducted using the DISCOVERY STUDIO v4.5 in Dock Proteins (ZDOCK) protocol that provides rigid body docking of two protein structures using the ZDOCK algorithm [40] as well as clustering the poses according to the ligand position. Optionally, the docked poses can be filtered for complexes that include a user specified set of residues at the binding interface. Z-dock score is the shape complementarity score calculated by the ZDOCK program and Z-rank score is the energy of the docked posed calculated by the ZRANK rescoring method, both of which were used to evaluate the best pose. The dockings were carried out in 3600 poses, screened out into 2000 better poses, and classified into 60 clusters according to RMSD = 0.6. Given that the Z-dock scores, Z-rank scores and the structures, we chose the most suitable poses of these complexes (BTLA + P0, BTLA + DP2, BTLA + P0-E1, BTLA + DP2-E1, BTLA + P0-E2 and BTLA + DP2-E2) with the Z-dock scores found to be 9.78, 13.08, 13.74, 11.62, 15.54, and 15.58, respectively.

### 3.6. Molecular Dynamics

To justify whether the models constructed from protein–protein docking are reliable, the molecular dynamics (MD) simulations of BTLA with P0/DP2/P0-E2/DP2-E2 bound were performed. The AMBER14 force field was used for the MD simulation as implemented in the YASARA (YASARA Biosciences GmbH, Vienna, VIE, AUT, (accessed on 5 June 2021)) program. The MD simulation employed periodic boundary conditions, the particle-mesh Ewald method for the treatment of the long-range coulomb forces beyond a 8 Å cutoff. 0.9% NaCl (a mass fraction) was used. Rescale the cell such that residues named HOH reach a density of 0.997 g/mL. No restraints were applied during the MD simulation using the settings employed in the second equilibration dynamics. The energies and coordinates every 100 ps were saved with a total simulation length of 100 ns at constant temperature (298 K) and pressure uncontrolled in NVT ensemble. Structural stability of the receptor–ligand complex was examined by analyzing the average values of potential energy with root mean square deviation (RMSD) throughout the trajectory. The RMSD profiles of all MD structures (Figure S4) shows that the variation of the RMSD values tends to be stable (<1 Å) after 60 ns, which means that the equilibrium structures have been obtained and the last MD structures can be chosen as representative ones from the most populated cluster.

## 4. Conclusions

Based on the co-crystal structure of BTLA/HVEM, we selected the core sequence on the binding interface from HVEM and conducted the saturation mutagenesis in silico. According to the results, 12 candidate peptides were synthesized and examined by competitive ELISA. Five of them (SP3, SP6, SP7, DP2, and DP3) showed inhibition. Further characterization revealed that the double mutant, DP2, is the most potent for blocking BTLA/HVEM interactions. To improve its efficacy, extended peptides based on DP2 were designed and synthesized, generating a peptide inhibitor with *K_D_* of nanomolar. Molecular docking of BTLA with different length of wild-type and mutated peptides revealed the mechanism of actions of the peptides. The two mutations E27P and E38F helped the short peptide (13 amino acids) stick on the interface of BTLA/HVEM. For the long one with 26 amino acids, the mutations caused a conformation change to form more hydrogen bonds with BTLA. In sum, we performed virtual evolution to the natural core fragments from HVEM in silico and screened out a good inhibitor–DP2. The peptide inhibitor was optimized by extending its sequence and its binding mechanism was elucidated by docking. The peptides could be potential drug candidates for immune therapy against cancers and the mechanism may provide some insights for further drug development.

## Figures and Tables

**Table 1:** Sequences of HVEM (26–38) mutants for screening.

Peptides	Amino Acid Sequence
P0-HVEM (26–38)	Ac-KEACGELTGTVCE-NH_2_
SP1 E27K	Ac-KKACGELTGTVCE-NH_2_
SP2 E27W	Ac-KWACGELTGTVCE-NH_2_
SP3 E27P	Ac-KPACGELTGTVCE-NH_2_
SP4 C29F	Ac-KEAFGELTGTVCE-NH_2_
SP5 C29Y	Ac-KEAYGELTGTVCE-NH_2_
SP6 E31D	Ac-KEACGDLTGTVCE-NH_2_
SP7 T33P	Ac-KEACGELPGTVCE-NH_2_
SP8 T35W	Ac-KEACGELTGWVCE-NH_2_
SP9 E38F	Ac-KEACGELTGTVCF-NH_2_
DP1 E27W E38F	Ac-KWACGELTGTVCF-NH_2_
DP2 E27P E38F	Ac-KPACGELTGTVCF-NH_2_
DP3 E27K E38F	Ac-KKACGELTGTVCF-NH_2_

**Table 2:** Binding affinity of the native and DP2 based mutated peptides with BTLA.

Peptides	*K_D_* (M)
P0	216.8
P0-E1	9.93 × 10^−3^
P0-E2	9.41 × 10^−5^
DP2	1.75 × 10^−3^
DP2-E1	3.04 × 10^−3^
DP2-E2	1.46 × 10^−9^

## Data Availability

Data is contained within the article or Supplementary Materials.

## Supplementary Materials

The following are available online at https://www.mdpi.com/article/10.3390/ijms22126638/s1.

## Abbreviations

BTLA	B- and T-lymphocyte attenuator
CTLA-4	Cytotoxic T-lymphocyte antigen-4
ELISA	Enzyme-linked immunosorbent assay
HVEM	Herpes virus entry mediator
PBS	Phosphate buffer saline
PD-1	Programmed cell death 1
PDB	Protein data bank
PD-L1	Programmed cell death-ligand 1
PPI_S_	Protein-protein interactions
SPPS	Solid-phage peptide synthesis
SPR	Surface plasmon resonance
