# Supplementary material for: Virtual Evolution of HVEM Segment for Checkpoint Inhibitor Discovery
Source: Int J Mol Sci. 2021 Jun 21;22(12):6638. doi: 10.3390/ijms22126638 (PMC8234244; doi:10.3390/ijms22126638)
Supplement: Supplementary file 1 [file ijms-22-06638-s001.zip › ijms-1197192-supplementary_2.pdf]

Supplementary Materials

Virtual evolution of HVEM segment for checkpoint inhibitor discovery

Mingjia Yu [1,†], Huimin Zhao [2,†], Yuhui Miao [2], Shi-Zhong Luo [2,*] and Song Xue [2,*]

1 Beijing Advanced Innovation Centre for Soft Matter Science and Engineering, Beijing University of Chemical Technology, Beijing 100029, China; 2019750009@mail.buct.edu.cn
2 Beijing Key Laboratory of Bioprocess, College of Life Science and Technology, Beijing University of Chemical Technology, Beijing 100029, China; 18634404469@163.com (H.Z.); ukimiao@foxmail.com (Y.M.)
* Correspondence: luosz@mail.buct.edu.cn (S.-Z.L.); xues@mail.buct.edu.cn (S.X.)
† Those two authors contributed equally to this article.

Principles of single point saturation mutagenesis *in silico*

The simulations of single point saturation mutagenesis were performed with CHARMm Polar H force field in pH-dependent mode. The pH value, ionic strength, solvent dielectric constant and energy cutoff were set at 7.4, 0.1, 80 and 0.5, respectively. The advanced parameters set for the simulations including electrostatic, van der Waals and entropy were all kept by default. The description for Calculate Mutation Energy (Binding) protocol will be illustrated in details as follows.

The Calculate Mutation Energy (Binding) protocol evaluates the effect of mutations on the binding affinity of molecular partners in protein-protein and protein-ligand complexes. It performs combinatorial amino-acid scanning mutagenesis on a set of selected amino-acid residues by mutating them to one or more specified amino-acid types. All energy terms are calculated by CHARMm and the electrostatics energy is calculated using a Generalized Born implicit solvent model. The energy effect of each mutation on the binding affinity (mutation energy, $\Delta\Delta G_{mut}$) is calculated as the difference between the binding free energy in the mutated structure and wild type protein:

$$\Delta\Delta G_{mut} = \Delta\Delta G_{bind}(\text{mutant}) - \Delta\Delta G_{bind}(\text{wild type})$$

The binding free energy, $\Delta\Delta G_{bind}$, is defined as the difference between the free energy of the complex and unbound state:

$$\Delta\Delta G_{bind} = \Delta G_{cmplx}(\text{complex}) - \Delta G_{unbnd}(\text{unbound state})$$

The calculations were performed in pH-dependent mode by using a model with titratable acidic and basic residues. The calculations of non-polar contributions to the free energy of binding are combined with calculations of protein ionization of the wild type and mutated structures in both the bound and unbound states. The electrostatic energy terms are obtained by integration over the proton binding isotherms, derived from the fractional protonation of the sites of titration. This model also includes the effect of ionic strength, I, on the calculated free energy terms. The total energy is calculated as an empirical weighted sum of van der Waals (E_{vdW}) interaction, electrostatic interactions (ΔG_{elec}), an entropy contribution ($-TS_{sc}$) related to the changes in side-chain mobility, and a non-polar, surface dependent, contribution to solvation energy (ΔG_{np}).

$$\Delta G_{tot}(pH) = aE_{vdW} + \Delta G_{elec}(pH,I) - cTS_{sc} + \Delta G_{np}$$

In this mode, the temperature changes are not taken into account and the results

correspond to temperatures at or close to 20°C.

Ref:

Spassov VZ, Yan L. pH-selective mutagenesis of protein-protein interfaces: in silico design of therapeutic antibodies with prolonged half-life. Proteins. 2013 Apr;81(4):704-14. doi: 10.1002/prot.24230. Epub 2013 Jan 15. PMID: 23239118; PMCID: PMC3601434.

ZDOCK method

ZDOCK is a rigid-body docking program that requires minimal information about the binding site and is targeted at initial-stage unbound docking. The program uses individual protein structures determined by experimental or computational methods as inputs and predicts the structure of a number of protein complexes (i.e., the top 2000 complexes). ZDOCK uses a simple shape complementarity method called Pairwise Shape Complementarity (PSC). The PSC method is optionally augmented with desolvation (DE) and electrostatic (ELEC) energy terms to rank the docked poses. PSC is not based explicitly on protein surface curvature or surface area, but rather rewards all close atomic contacts between the protein receptor and the protein ligand within a specific cutoff distance. PSC has been shown to yield better results than the common grid-based shape complementarity (GSC) method. It rewards contiguous surface patches at the binding site and implicitly accounts for the curvature of the binding surface.

Ref:

Chen R, Weng Z. A novel shape complementarity scoring function for protein-protein docking. Proteins. 2003 May 15;51(3):397-408. doi: 10.1002/prot.10334. PMID: 12696051.

Chen R, Li L, Weng Z. ZDOCK: an initial-stage protein-docking algorithm. Proteins. 2003 Jul 1;52(1):80-7. doi: 10.1002/prot.10389. PMID: 12784371.

Yes, the details of the docking calculation are given in the supplementary material, which include all the scores of top ranked protein poses.

Figure S1. SPR results of the mutated and native peptides with BTLA.

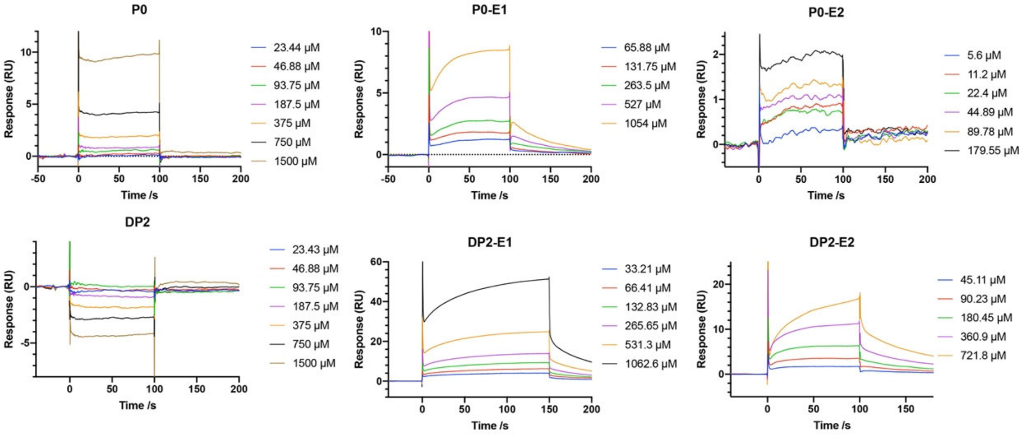

Figure S2. ZDOCK scores of top ranked protein poses and the chosen one.

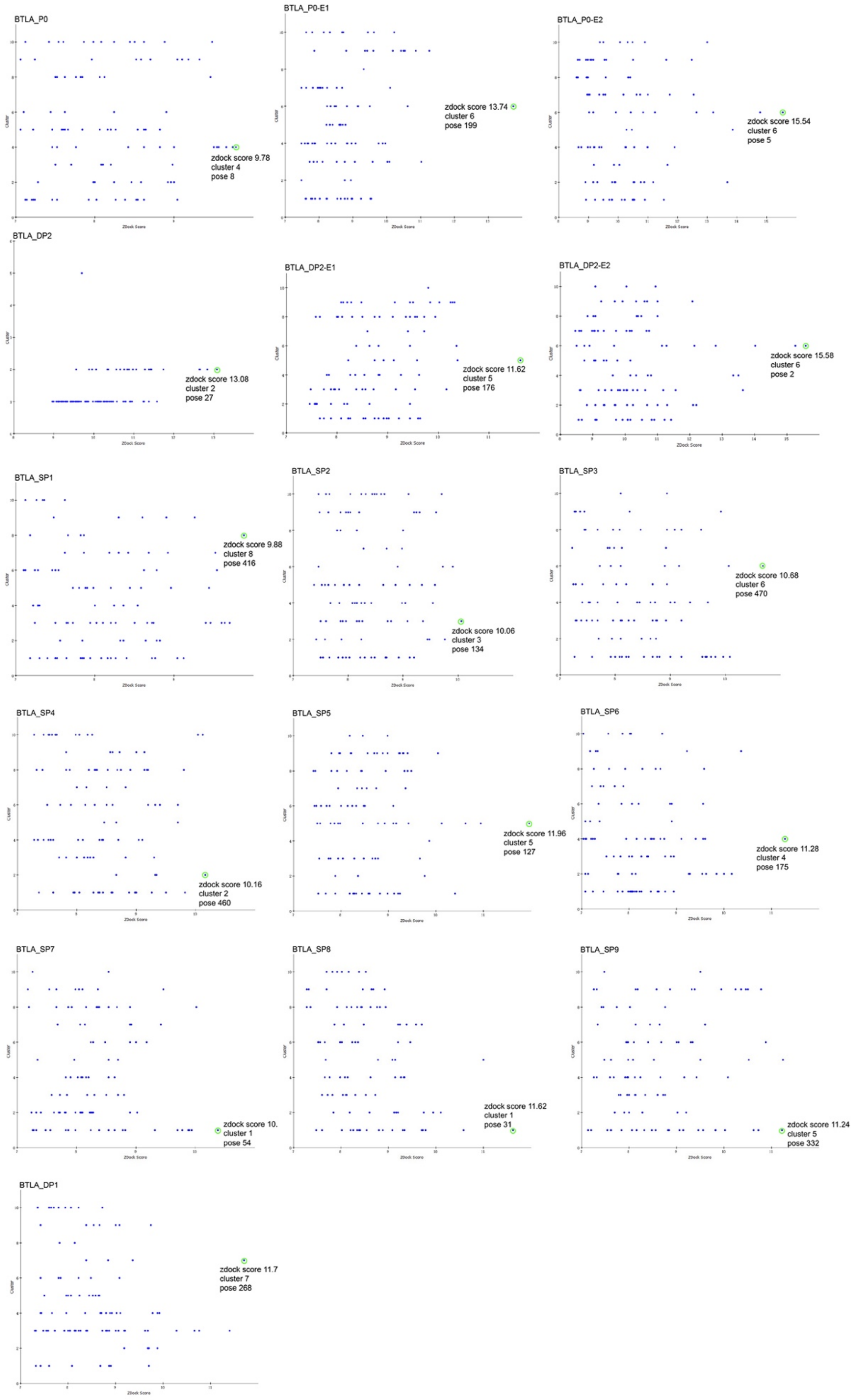

Figure S3. Superposition of two complexes including the last structure of BTLA+P0/BTLA+DP2/BTLA+P0-E2/BTLA+DP2-E2 obtained from the MD (colored in yellow) and the complex of BTLA+P0/BTLA+DP2/BTLA+P0-E2/BTLA+DP2-E2 obtained from zdock complex (colored ice blue). The RMSD values of the structures aligned by PyMOL including the last structure of BTLA+P0/BTLA+DP2/BTLA+P0-E2/BTLA+DP2-E2 obtained from the MD (colored in yellow) and the complex of **(A)** BTLA+P0/**(B)** BTLA+DP2/**(C)** BTLA+P0-E2/**(D)** BTLA+DP2-E2 obtained from zdock complex (colored ice blue) are 0.937, 0.902, 0.824 and 0.639 respectively.

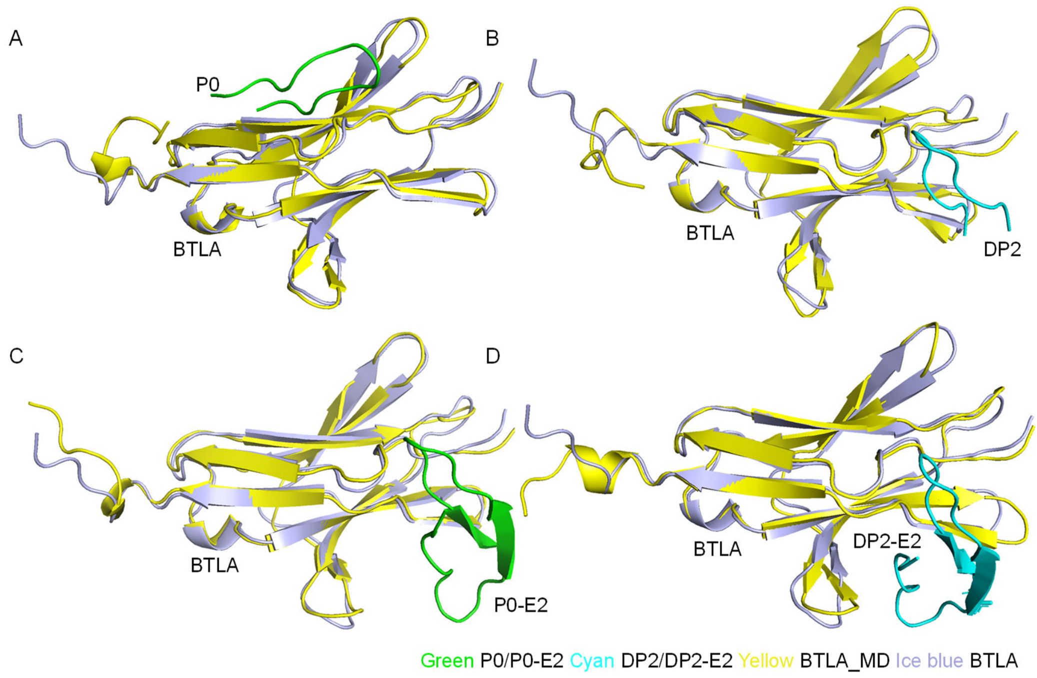

Figure S4. The RMSD profiles of 100 ns MD simulations of BTLA with P0/DP2/P0-E2/DP2-E2. The RMSD values of complexes between (A) BTLA and P0, (B) BTLA and DP2, (C) BTLA and P0-E2, (D) BTLA and DP2-E2. The RMSD values of Calpha [RMSDCa], backbone [RMSDBb] and all-heavy atom [RMSDAll] are shown in red, blue and green, respectively.

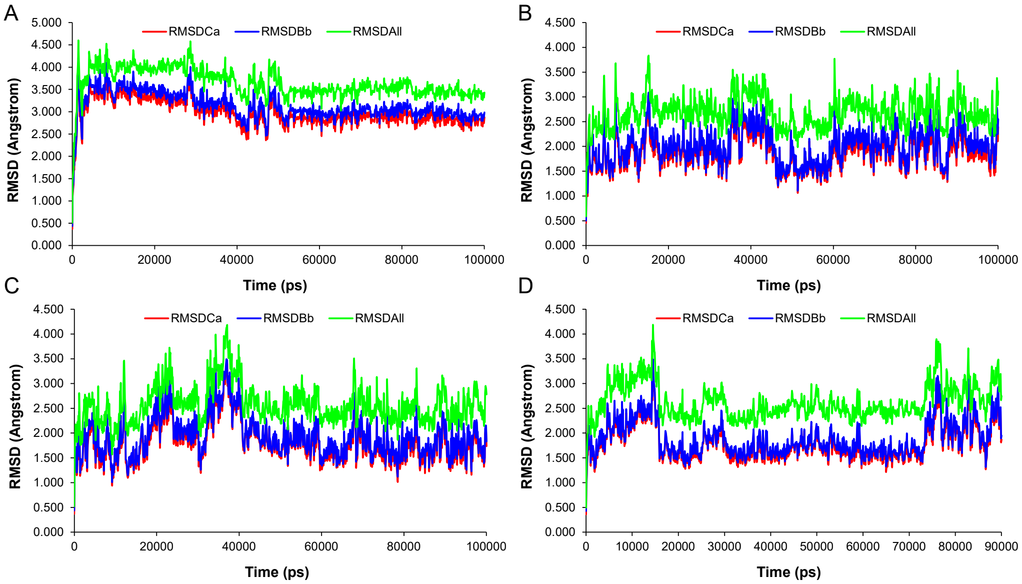

Table S1. The interaction of hydrogen bonds between BTLA and DP2.

Atoms in the Residues of BTLA	Atoms in the Residues of DP2	Hydrogen bond (Å)	Hydrogen bond angle (°)
GLN37/HE21	GLY34/O	2.2101	160.549
ARG42/HN	GLU31/O	1.8424	141.57
ARG42/HN	GLU31/OE2	2.464	92.077
ARG42/HE	GLU31/OE1	2.4198	132.089
LEU123/HN	CYS37/O	2.0297	93.175
GLU125/HN	THR35/O	2.3129	121.307
LEU38/O	GLY34/HN	2.1359	161.433
GLU125 /O	THR35/HN	2.1144	110.694
LEU123 /O	CYS37/HN	2.0735	119.407
LYS41/HA	GLU31/O	2.0351	117.897
LYS41/HE1	GLU31/OE2	2.5038	122.423
ILE124/HA	THR35/O	1.4613	155.202
ILE40/O	LEU32/HA	2.0928	106.02
GLU125/O	GLY34/HA1	2.7331	129.114
GLU125/O	GLY34/HA2	2.7516	95.408

Table S2. The interaction of hydrogen bonds between BTLA and DP2-E1.

Atoms in the Residues of BTLA	Atoms in the Residues of DP2-E1	Hydrogen bond (Å)	Hydrogen bond angle (°)
ARG42/HH12	CYS29/O	3.0172	90.32
GLY76/HN	CYS37/O	2.2678	135.086
GLU125/O	GLY34/HN	2.3041	95.5
GLY76/HA1	CYS37/O	2.5818	97.088
SER128/HB2	THR35/O	2.2794	132.316
GLN37/OE1	LEU32/HA	2.8345	112.242
GLU125/O	THR33/HA	1.7056	143.779

Note: The highlighted indicate unique amino acids contributing to the interactions between BTLA and each peptide.

Table S3. The interaction of hydrogen bonds between BTLA and P0-E2.

Atoms in the Residues of BTLA	Atoms in the Residues of P0-E2	Hydrogen bond (Å)	Hydrogen bond angle (°)
GLN37/HE21	GLU27/OE2	2.8517	150.665
GLN37/HE22	GLY34/O	2.6915	95.625
ARG42/HN	GLU31/O	1.7763	136.46
ARG42/HH22	SER15/O	3.0938	141.158
LEU123/HN	CYS37/O	1.7549	123.326
GLU125/HN	THR35/O	1.9064	144.883
GLU125/OE2	SER20/HG	3.0083	151.178
GLU125/O	THR35/HN	1.8262	143.176
LEU123/O	CYS37/HN	1.7289	126.044
ASN122/HA	CYS37/O	2.7438	128.288
ILE124/HA	THR35/O	1.9477	141.004
HIS127/HD2	THR33/O	2.7022	108.008
GLU125/O	GLY34/HA2	2.7293	97.188
SER121/O	PRO39/HD2	2.8154	97.201

Note: The highlighted indicate unique amino acids contributing to the interactions between BTLA and each peptide.

Table S4. The interaction of hydrogen bonds between BTLA and DP2-E2.

Atoms in the Residues of BTLA	Atoms in the Residues of DP2-E2	Hydrogen bond (Å)	Hydrogen bond angle (°)
ARG42/H	GLU31/O	1.6701	173.131
ARG42/HE	GLU31/OE1	1.7993	131.64
ARG42/HH21	SER15/O	2.5585	158.873
ARG114/HH12	LYS18/O	1.8617	129.131
LEU123/HN	CYS37/O	1.8176	148.977
GLU125/OE2	SER20/HN	2.5879	153.177
GLN37/OE1	GLY34/HN	2.4337	143.067
LEU38/O	GLY34/HN	2.5105	136.492
TYR39/HA	LEU32/O	2.1072	146.878
LYS41/HA	GLU31/O	2.6557	125.724
LYS41/HE1	GLU31/OE2	2.6222	137.676
ILE124/HA	THR35/O	2.366	102.526
GLU125/OE2	CYS19/HA	2.658	116.452
ILE40/O	LEU32/HA	2.8481	104.713
LEU38/O	THR33/HA	2.0442	162.812
GLU125/O	GLY34/HA2	2.0472	99.317
GLU125/O	THR35/HB	3.074	105.808
LEU123/O	VAL36/HA	1.844	120.687
SER121/O	PRO39/HD2	2.5245	165.199

Note: The highlighted indicate unique amino acids contributing to the interactions between BTLA and each peptide.
